# Chemical and Thermal Characteristics of Ion-Exchanged Lignosulfonate

**DOI:** 10.3390/molecules28062755

**Published:** 2023-03-18

**Authors:** Eko Setio Wibowo, Byung-Dae Park

**Affiliations:** 1 Department of Wood and Paper Science, Kyungpook National University, Daegu 41566, Republic of Korea; 2 Research Center for Biomass and Bioproducts, National Research and Innovation Agency, Bogor 16911, Indonesia

**Keywords:** lignosulfonate, ion-exchange, kraft lignin, lignin structure, thermal properties

## Abstract

Lignosulfonate features sulfonate groups, which makes it soluble in water and hence, suitable for a wide range of applications. However, its characterization is challenging because of its limited solubility in organic solvents. Thus, this study investigated the chemical and thermal characteristics of ion-exchanged sodium lignosulfonate (Na-LS) and compared it with those of industrial kraft lignin derived from softwood and hardwood. The results demonstrated that the ion exchange successfully converted Na-LS to lignosulfonic acid (H-LS), as proven by the Fourier-transform infrared spectroscopy (FTIR), X-ray photoelectron spectroscopy (XPS), and elemental analysis. H-LS has a greater apparent molecular weight than those of Na-LS and softwood and hardwood kraft lignin (SKL and HKL). According to ^31^P nuclear magnetic resonance (NMR) analysis, H-LS has less phenolic OH than SKL and HKL, indicating that it has more polymeric chains. Furthermore, H-LS has substantially more native side chains, such as β-*O*-4 units, than SKL and HKL. Thermal analysis revealed that H-LS has a greater glass temperature (T_g_) than SKL and HKL, although Na-LS has a lower T_g_ than SKL and HKL. In addition, H-LS degraded faster than Na-LS did because the acid condition accelerated degradation reaction.

## 1. Introduction

The chemical pulping industry currently produces around 70 million tons of lignin per year as a byproduct with only 5% commercial use, and the rest is used as an energy source [1,2,3]. Lignosulfonate is one of the industrial lignin extracted from the black liquor of the sulfite pulping process and is primarily utilized for a variety of applications, such as wood adhesive [4], dispersant [5,6], and even antioxidant [7]. This is because, unlike other industrial lignin such as kraft lignin, which has poor water solubility, the sulfonic groups attached primarily at the C_α_ position during sulfite pulping allow lignosulfonate to be soluble under neutral or moderately acidic conditions [7,8]. Furthermore, lignosulfonic acid (H-LS), the acid form of lignosulfonate, can offer a significant amount of the sulfonic acid group, which can be deployed as a proton transfer agent for catalytic activity and stimulate a wide range of reactions [9,10,11]. Due to the numerous applications of lignosulfonate, the chemical characteristics of isolated lignosulfonate, such as the identification of total OH groups and lignin side chains, are crucial. For example, total phenolic/aromatic OH information is vital for antioxidant activity [12] and wood adhesive applications [13], whereas large amounts of sulfonic acid groups are essential for catalytic activity. However, the main challenge with lignosulfonate characterization is its poor solubility in organic solvents due to the strong polar sulfonic groups present. As a result, a suitable method for lignosulfonate characterization is necessary.

Furthermore, it has been reported that the derivatization of lignin via the phosphitylation reaction and the ^31^P nuclear magnetic resonance (NMR) technique is the most accurate method for determining total aliphatic, aromatic, and carboxylic OH [14]. Due to the use of chloroform and pyridine as the main solvents for this procedure, which scarcely dissolve lignosulfonate, researchers have developed new methods for estimating the total OH of lignosulfonate. For example, Korntner et al., provided three alternative methods of determining OH groups in lignosulfonate [15]. These procedures comprised ultrasonication (160 W, 30 °C) for 1 h, suspending in *N*,*N*-dimethylformamide (DMF) solvent before ultrasonication (160 W, 30 °C) for 15 min, and pre-swelling in dimethylsulfoxide (DMSO). These ultrasonication pre-treatment approaches resulted in the complete dissolution of lignosulfonate sample; nevertheless, this procedure carries the risk of polymer degradation [15,16]. Korntner et al. even noticed a massive increase in aliphatic OH groups after 1 h of sonication, indicating that ether or ester bonds were ruptured, releasing OH groups [15]. They concluded that pre-swelling in DMSO was the preferable method, but they also highlighted that DMSO was chemically incompatible with the derivatization reagent. Recently, Stücker et al. have suggested a new and simple approach for determining OH groups in lignosulfonates [17]. This method involved ion-exchanging lignosulfonate to produce H-LS, which was then lyophilized and dissolved in a combination of anhydrous DMF, deuterated DMF, and pyridine (4.5:1:1; *v/v*). The conventional relaxation agent, phosphitylation reagent, and internal standard from the established method [14] were also included.

This study adopted the ion-exchange method to quantify the total OH groups of industrial sodium lignosulfonate (Na-LS), using ^31^P NMR spectroscopy after it was converted to H-LS. Subsequently, the H-LS sample was analyzed using the Fourier-transform infrared (FTIR) spectroscopy, an elemental analyzer, X-ray photoelectron spectroscopy (XPS), gel permeation chromatography (GPC), and ^1^H–^13^C heteronuclear single quantum coherence (HSQC) NMR to determine the functional groups, elemental composition, apparent molecular weight, and lignin side chains. In addition, differential scanning calorimetry (DSC) and thermogravimetric (TG) analyses were utilized to evaluate and compare the thermal characteristics of Na-LS and H-LS samples, providing useful information for further applications.

## 2. Results and Discussion

### 2.1. Ion-Exchanged Lignosulfonate

**Figure 1:**
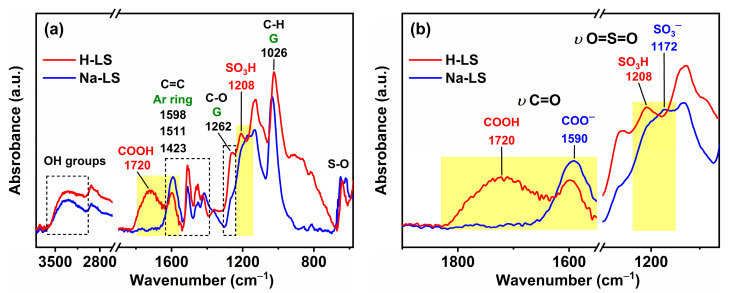
FTIR spectra of lignosulfonate samples (Na-LS and H-LS): (**a**) entire FTIR spectra, (**b**) enlarged FTIR spectra at a selected region (marked by yellow color).

**Scheme 1:**
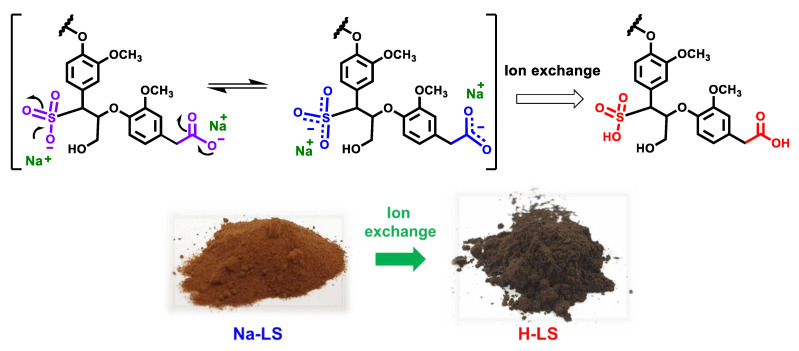
Chemical conversion and color change of Na-LS to H-LS using ion exchange chromatography.

Figure 1a shows a comparison of the FTIR spectra of the Na-LS and H-LS samples. It shows that the functional groups in the Na-LS and H-LS samples are relatively similar. For example, both share a similar aromatic skeleton vibration (C=C bonds) of lignin (1598 cm^–1^, 1511 cm^–1^, and 1423 cm^–1^) and C–H bending vibrations of –CH_2_– and –CH_3_ at around 1450 cm^–1^ [18,19]. Moreover, the C–O and C–H bands associated with the guaiacyl unit of lignin were detected in both samples at roughly 1262 cm^–1^ and 1026 cm^–1^ [18,19]. In addition tothat, the characteristic S–O band of lignosulfonates at roughly 650 cm^–1^ [20,21] was identified in both Na-LS and H-LS samples. Furthermore, the enlarged FTIR spectra in Figure 1b validated the conversion of Na-LS to H-LS by ion-exchange chromatography. Following the ion exchange process, two additional peaks at roughly 1720 cm^–1^ and 1208 cm^–1^ associated with COOH and SO_3_H were observed, indicating the formation of H-LS. The carbonyl ion (COO^–^, 1590 cm^–1^) in Na-LS has a lower wavenumber than the carbonyl of the carboxylic acid (COOH, 1720 cm^–1^) in H-LS. This is due to the negative charge of the ionized carbonyl groups (COO^–^), which can resonate between two oxygen atoms [22] (see Scheme 1), weakening the C=O bond and decreasing the frequency of vibration (shift to lower wavenumber). It should be highlighted that the peak at around 1598 cm^–1^ also corresponds to the C=C vibration of the aromatic groups, as previously explained, and therefore, the Na-LS has a higher intensity around this wavenumber than the H-LS due to the overlapping band of COO^–^ and C=C of the aromatic ring (Figure 1b). A similar phenomenon occurred with the vibration of O=S=O. The characteristic vibration of O=S=O in H-LS (SO_3_H), which is mostly detected at around 1200 cm^–1^ [11,20], has a higher wavenumber than O=S=O in Na-LS (SO_3_^–^, 1172 cm^–1^). This is due to the resonance of the lone pair electrons in Na-LS, which weakens the O=S=O bonds (Scheme 1). In addition, the absorbance of O=S=O in SO_3_^–^ (ammonium and sodium salts) has been reported to be 1173 cm^–1^ and 1180 cm^–1^, respectively [21], which is in agreement with the results of this work.

Scheme 1 depicts the chemical conversion of Na-LS to H-LS, as well as the color shift from light brown to dark brown. The color darkened due to the conversion of –COONa or –SO_3_Na to –COOH or –SO_3_H, which increased the hydrogen between lignin molecules. This hydrogen bonding interaction promoted lignin macromolecule aggregation and created a tighter link between lignin clumps [23]. As a result, lignin’s bulk density increased. This causes the lignin chromophore to become more concentrated on a macroscopic scale, resulting in a darker lignin color [23]. In contrast, although the Na-LS comprises several OH groups that may potentially form hydrogen bonds with lignin polymers, the excess of negative charges in Na-LS inhibits aggregation due to electrostatic repulsion, giving the Na-LS a lighter color. It has been reported that incorporating a sulfonate or sulfate group can cause polymer aggregation owing to an increase in inter and intramolecular interactions [24].

Table 1 demonstrates the differences in various parameters, such as elemental composition and ash content, between Na-LS and H-LS samples. The chemical element composition (C, H, N, S, and O) of these samples is similar to that reported in the literature [21,25,26,27]. Na-LS appears to be a better candidate for energy generation than H-LS. This is due to the fact that the high carbon and low oxygen content (low O/C ratio) are favorable for energy-oriented application, since the lower O/C ratio results in a higher calorific value [28]. In general, H-LS has a greater elemental percentage than Na-LS due to the high ash content in Na-LS, which reduces the proportion of its chemical element. The high ash percentage of Na-LS (~18%) is comparable with those reported in the literature for Ca-LS (~15%) and Na-LS (>20%) [17,26,27]. In contrast, H-LS had a low ash percentage (~1.4%). This result clearly reveals a large decrease in ash content (~92%) following ion exchange treatment. This occurred because a high ash content is associated with a large amount of metal ions, such as sodium, calcium, and other inorganic substances. Thus, the ion exchange treatment on Na-LS was shown to efficiently reduce ash content while also removing other contaminants [17]. Furthermore, the carbon content of lignosulfonate samples (Na-LS and H-LS) is approximately 42–48%, which is relatively low in contrast to SKL [29] and HKL [29,30], which is around 60%. Low carbon content appears to be prevalent for lignosulfonate [21]. Moreover, low nitrogen amounts were discovered in these lignosulfonate samples, which is likely attributable to the presence of organic compounds, such as proteins, some of which may have originated from the raw material [5,25,31]. Furthermore, the sulfonate group calculated from the sulfur content was about 2 mmol/g, which is somewhat higher than that reported elsewhere (1.97 mmol/g) [11]. It has been confirmed that a high sulfonation degree implies a better dispersant ability for cement and coal–water slurry [32], as it may improve the water solubility of lignin macromolecules owing to the introduction of additional negative charges. In addition to that, the high SO_3_H concentration in H-LS can be beneficial to acid catalytic activity [11].

**Figure 2:**
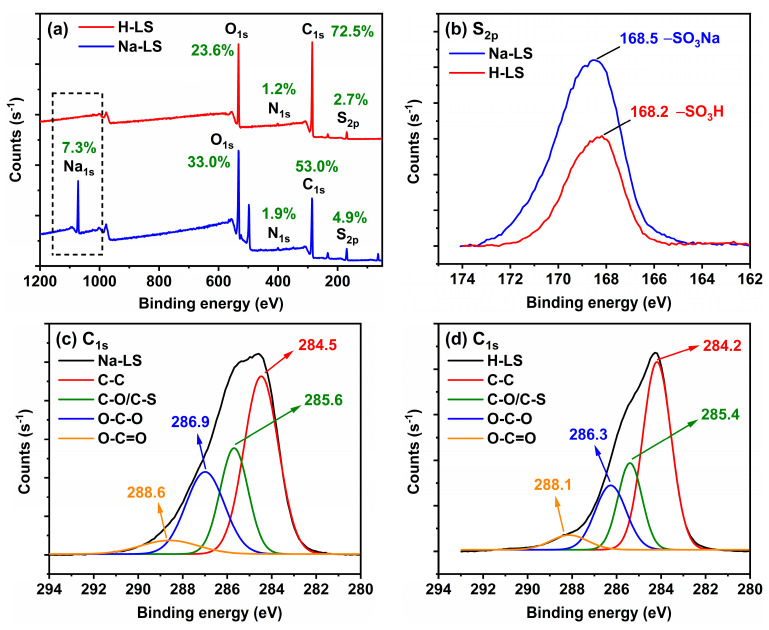
(**a**) XPS survey spectra of Na-LS and H-LS, (**b**) high-resolution S_2p_ spectra of Na-LS and H-LS, (**c**) high-resolution C_1s_ spectra of Na-LS, and (**d**) high-resolution C_1s_ spectra of H-LS.

The XPS survey spectra of Na-LS and H-LS samples are shown in Figure 2a. The chemical composition of the sample surfaces revealed that sodium metal was completely eliminated by the ion exchange, as seen by the disappearance of the Na_1s_ peak at ~1072 eV [33], resulting in a sodium content reduction from 7.3% to 0%. This finding confirms the FTIR and elemental analysis results, suggesting a successful conversion of Na-LS to H-LS by ion exchange treatment. In addition, the high-resolution S_2p_ spectra of Na-LS and H-LS (Figure 2b) revealed strong peaks at ~168 eV attributed to –SO_3_Na or –SO_3_H [33,34], confirming the existence of C–SO_3_Na or C–SO_3_H in lignosulfonate samples. Furthermore, Figure 2c,d show high-resolution C_1s_ spectra containing various chemical bonds associated with carbon atoms in lignosulfonate samples. Moreover, the most prominent peaks are those at 256–256 eV ascribed to C–O or C–S [34] and 288–289 eV assigned to COOH or COONa [33,34]. The presence of these peaks suggested that lignosulfonate contains ether (C–O–C), alcohol (C–OH), sulfonate (C–SO_3_H/Na), and carboxylic (COOH/Na) bonds.

**Figure 3:**
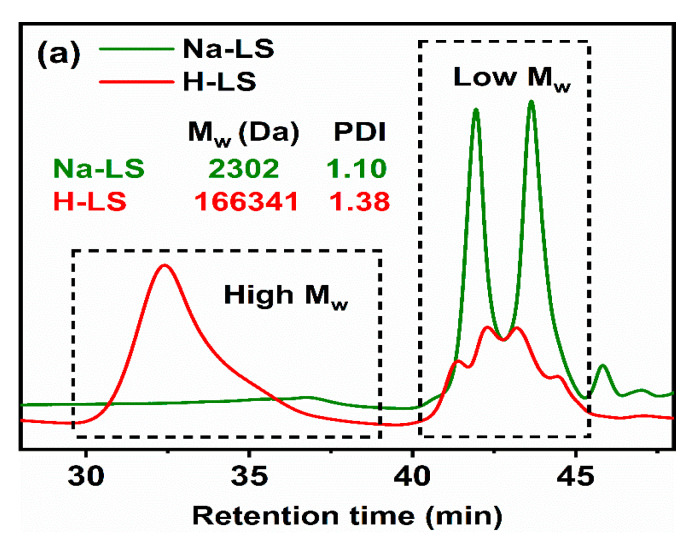

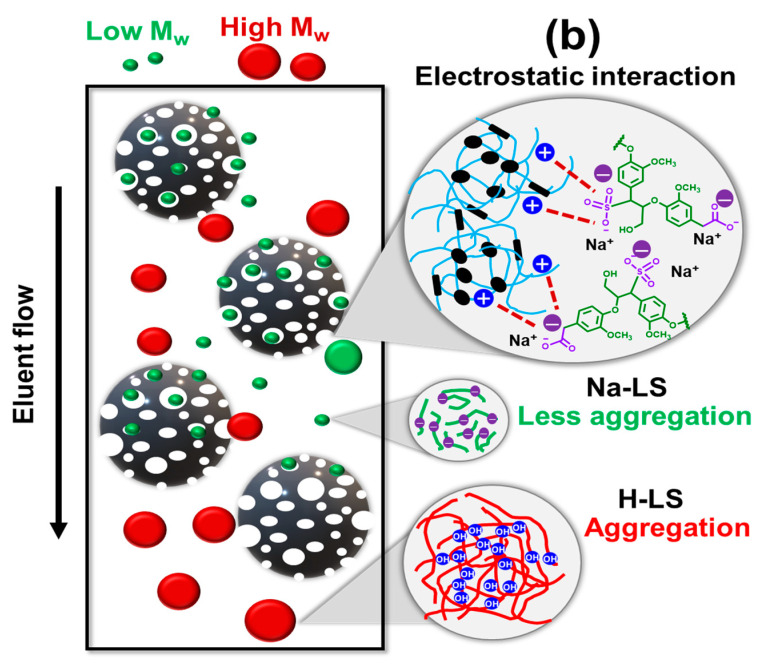
(**a**) GPC chromatogram of lignosulfonate samples (Na-LS and H-LS), as well as their apparent molecular weight and polydispersity. (**b**) An illustration of the electrostatic interaction of charged lignosulfonate with a column material.

**Scheme 2:**
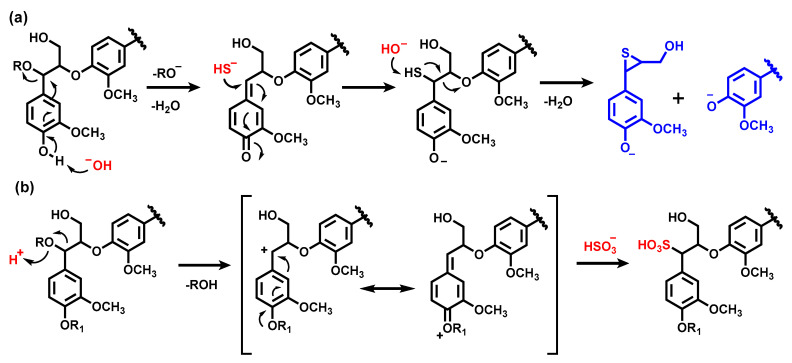
Chemical reactions occurred during (**a**) kraft pulping (cleavage of the β-*O*-4′ linkage) and (**b**) sulfite pulping (sulfonation reaction). Chemical reactions were adapted from Demuner et al. (2019) [40].

Figure 3a depicts GPC thermograms of Na-LS and H-LS samples. H-LS samples clearly outperformed Na-LS in the low retention time region (high M_w_). On the other hand, prominent and sharp peaks of the Na-LS sample were found at the high retention time region (low M_w_). Consequently, the apparent M_w_ of H-LS was substantially greater than that of Na-LS. This is because the Na-LS salt solution may contain more negative charges than the H-LS solution, which may interfere with the GPC measurement. It has been suggested that the lignosulfonate polymer is a randomly branched polyelectrolyte [35], allowing electrostatic interaction of Na-LS with the column material (ultrahydrogel) and, therefore, influencing the results of GPC measurement [17] (see Figure 3b). For example, high M_w_ molecules should be eluted quickly or have a lower retention time, while low M_w_ molecules may instead have a longer retention time because of the electrostatic attraction with the column material (Figure 3b). Moreover, a rise in apparent M_w_ has been observed as a response to the charge alteration, which causes rearrangements in the aggregation state [17]. Na-LS with an abundance of negative charges can produce less aggregation between lignin molecules, whereas H-LS with fewer negative charges can cause more aggregation owing to hydrogen bonding between lignin molecules (Figure 3b). High M_w_ values, such as in H-LS, appear to be more accurate, as large apparent M_w_ values are typical in lignosulfonates samples [36,37,38]. However, it is generally accepted that aqueous GPC analysis of lignosulfonates is extremely complex, and the results can be difficult to interpret because common calibration standards are frequently not identical to the hydrodynamic volume of the actual lignin sample due to differences in shape and extensions [38,39]. Thus, molar masses of lignin samples should be regarded as approximations or relative and may not accurately represent the lignin’s molecular weight. Additionally, the apparent M_w_ of H-LS samples was greater than that of SKL (8725 Da) and HKL (3982 Da) [29]. This is because lignin has been depolymerized into smaller fragments during the kraft pulping process by breaking the lignin linkages, particularly the β-*O*-4′ linkage (see Scheme 2a) so that it could be soluble in the cooking liquor [40]. By contrast, the main reason for lignin dissolution in sulfite pulping is the introduction of polar sulfonate groups (sulfonation) at the carbon-α position by, first, breaking the α-*O*-4′or hydroxyl groups bonded at such a position [40] (see Scheme 2b). Therefore, lignosulfonate generally has greater molecular weights than kraft lignin because only a small portion of the most abundant ether bond in lignin, β-*O*-4′ linkage, is degraded during the pulping process [40].

### 2.2. Chemical Structure of Ion-Exchanged Lignosulfonate

**Figure 4:**
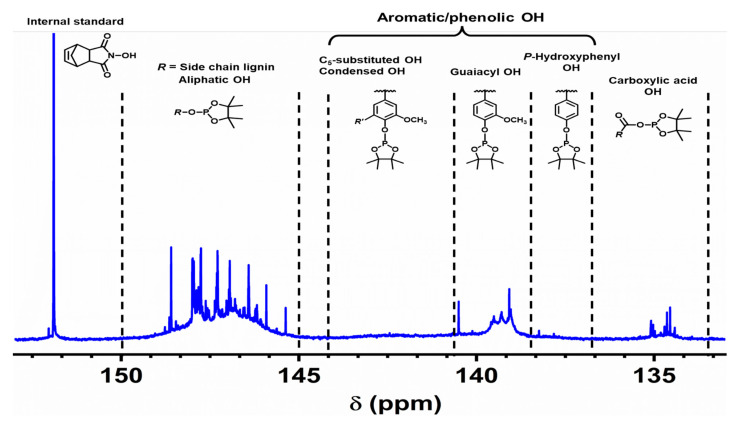
^31^P NMR spectra of lignosulfonic acid (H-LS) after phosphitylation with TMDP.

To fully understand its characteristics and provide useful knowledge for future applications, the chemical structure of lignosulfonate should be thoroughly investigated. Figure 4 displays the ^31^P NMR spectra of an H-LS sample after derivatization. This ^31^P NMR spectra were clearly separated into various regions that contain various kinds of hydroxyl groups, such as aliphatic, aromatic, and carboxylic acid hydroxyl groups. When compared with the aromatic and carboxylic OH, H-LS showed the most intensive peak of aliphatic OH groups, indicating that the lignosulfonate sample contains more aliphatic OH than aromatic OH. However, it is noticeable that the aliphatic OH peak was not uniform but rather overlapped with numerous small and sharp peaks, suggesting that some aliphatic OH may not belong to lignin structures. One of the most suspicious reasons is the presence of carbohydrate impurity [26] since carbohydrates contain aliphatic OH groups having chemical shifts at approximately 145–149 ppm [41]. Additionally, because the lignosulfonate was derived from softwood, the syringyl signal was scarcely detectable in the aromatic OH area of ^31^P NMR spectra.

Table 2 presents the quantitative results of the hydroxyl content acquired by integrating the area under the curve and compares the values to the internal standard. The hydroxyl content of the lignosulfonate sample agrees with the hydroxyl content of softwood lignosulfonate published in the literature [17]. Interestingly, the amount of aromatic/phenolic OH groups in lignosulfonate samples was significantly lower than those of SKL and HKL samples. This is because lignin is not extensively degraded in sulfite pulping as it is in kraft pulping. The cleavage of the β-*O*-4′ linkage in kraft pulping might result in a substantial amount of phenolic OH (see Scheme 2a). Low phenolic OH groups in the lignosulfonate suggest that this lignin has more polymeric chains than kraft lignin because the hydrogen in the phenolic OH is substituted by chemical bonds with other units. As a result, H-LS exhibited predominantly the non-phenolic lignin structure, which was also compatible with greater M_w_ than SKL and HKL in the GPC data. Furthermore, the proportion of condensed phenolic OH groups in kraft lignin samples was higher than in lignosulfonate, particularly in the SKL sample. This is due to the harsh kraft pulping conditions (high temperature, high pH, and strong nucleophiles, HS^–^), which promotes retro aldol and radical coupling reactions, and leads to the formation of condensed structures like the 1–5ʹ and 5–5ʹ linkages [42], especially in SKL, which is abundant in guaiacyl units with free C5 positions.

**Figure 5:**
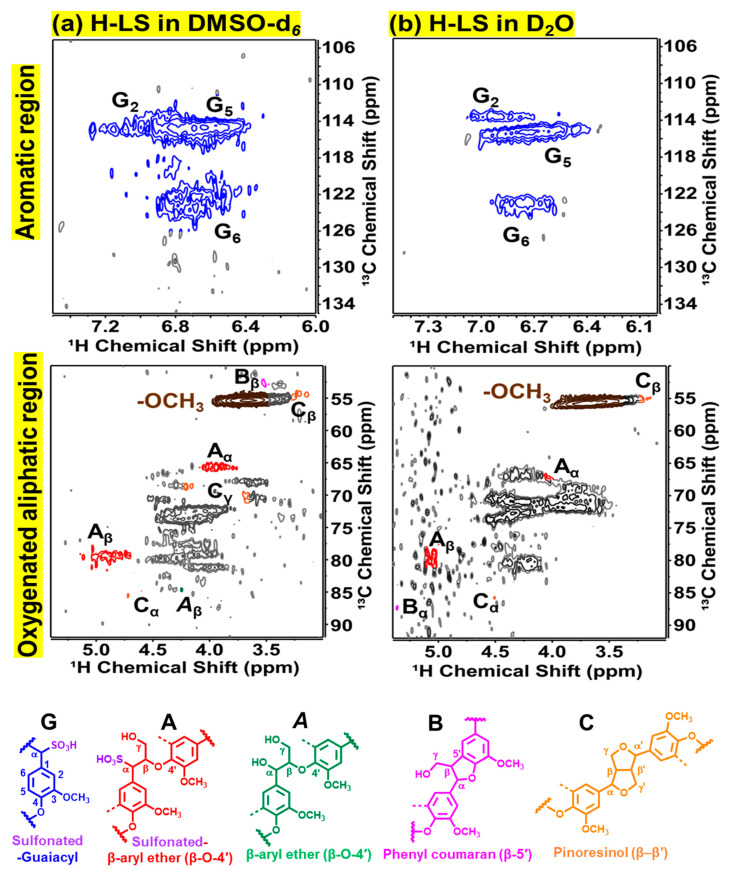
^1^H–^13^C HSQC NMR spectra of lignosulfonic acid (H-LS) sample recorded in different solvents: (**a**) DMSO-d_6_ and (**b**) D_2_O.

Figure 5 depicts 2D ^1^H–^13^C HSQC NMR spectra of an H-LS sample measured with different solvents (DMSO-d_6_ and D_2_O). The NMR spectra revealed two distinct regions: aromatic and oxygenated aliphatic. Signals associated to guaiacyl (G) units, such as G_2_, G_5_ and G_6_, were clearly observed in the aromatic region. Subsequently, in the oxygenated aliphatic region, signals associated to different lignin side chains, such as sulfonated β-*O*-4′, β-β, and β-5′, were identified. Surprisingly, the NMR spectra demonstrated that these two solvents exhibited considerably distinct patterns. For example, at the aromatic region, D_2_O solvent clearly separates the G_2_ and G_5_ signals, and yet DMSO-d_6_ solvent provided slightly overlapping G_2_ and G_5_ signal, which is fairly common for lignosulfonate [27,43,44,45]. Nevertheless, DMSO-d_6_ performed better in the oxygenated aliphatic region. The signals for different lignin units were clear, and there were less noises when dissolved in DMSO-d_6_ (Figure 5a) than D_2_O (Figure 5b). This is most likely owing to the ionization of sulfonate (SO_3_H) and carboxylate (COOH) groups in the D_2_O solvent, which might interfere with the NMR measurement. The enhanced conductivity of the samples due to the high salt content has been observed to decrease the performance of NMR cryoprobes [46]. Furthermore, the NMR spectra at the oxygenated aliphatic region revealed that the lignosulfonate sample contained a high concentration of impurities, as evidenced by complex signals at around δ_C_/δ_H_ 65–80/3.5–4.5 ppm, regardless of the solvent used. These NMR spectra with contaminants were similar to those observed in the literature [44,47]. As previously reported, these signals were mostly originated from the carbohydrate’s impurity attached to the lignosulfonate [44,47]. This is because lignin has not been selectively removed during the sulfite pulping process, and carbohydrates, notably hemicelluloses, are extensively solubilized in the cooking liquor [40]. Therefore, this liquor has high carbohydrate content as well as certain additional chemicals used in the pulping process. It is difficult to separate lignin and carbohydrates because they are extensively cross-linked and soluble in water [44], although several methods such as fermentation, membrane separation, and ultrafiltration are often employed to obtain pure lignosulfonate [40]. Remarkably, Khokarale et al. successfully purified the lignin fraction from carbohydrate impurities using a switchable ionic liquid (SIL) method under mild treatment conditions [44]. However, further purification to achieve carbohydrate-free lignin is not performed in this study because the main objective of this research is to examine the influence of ion exchange treatment on the chemical and thermal properties of industrial lignosulfonate.

**Scheme 3:**
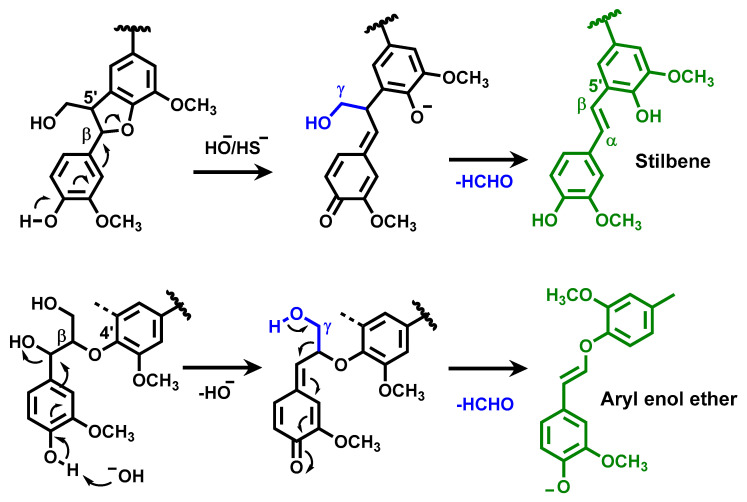
Chemical transformations occurred during kraft pulping. Adapted from Crestini et al. (2017) [49].

Table 3 demonstrates the relative abundance of side chain units in various lignin samples. As expected, the relative abundance of H-LS is consistent and equivalent to that of the softwood lignosulfonate reported in the literature [48]. Clearly, H-LS contained significantly more lignin side chain linkages, particularly sulfonated β-*O*-4′, than kraft lignin samples (SKL and HKL). This is due to differences in delignification during the pulping process. As explained previously, the lignin removal in sulfite pulping was mostly due to the incorporation of sulfonate groups into the lignin structure (Scheme 2b) rather than lignin depolymerization into smaller fragments by breaking the β-*O*-4′ linkage (Scheme 2a). The mechanisms involve the acid causing a hydroxyl group to be removed or the α-ether linkage to be cleaved, resulting in quinone methide intermediates through a resonance-stabilized benzylic cation [8]. Sulfur is then covalently bonded with lignin by connecting sulfonate groups (HSO_3_^–^) to an α-carbon (Scheme 2b). With the addition of the abundant sulfonate groups, lignin becomes hydrophilic, exhibiting increased water solubility and making it easier to eliminate from the pulp. The high concentration of sulfonated β-*O*-4′ linkage in the H-LS sample is consistent and supports the results of GPC and ^31^P NMR, which exhibit high apparent M_w_ and a low amount of aromatic OH (high polymeric chains). Correspondingly, the H-LS had more other side chain units than SKL and HKL, which was likely owing to a substantial breakdown of the kraft lignin or transformation into newly generated units [29,49]. During the kraft pulping, for example, phenyl coumaran (β-5′) and β-aryl ether (β-*O*-4′) might be converted into stilbene and aryl enol ether via a retro-aldol reaction activated at the γ-carbon OH and releasing formaldehyde [42] (see Scheme 3). The quantity of stilbene and aryl enol ether produced from the kraft lignin samples (SKL and HKL) can be seen in the previous study [29].

### 2.3. Thermal Behavior of Ion-Exchanged Lignosulfonate

**Figure 6:**
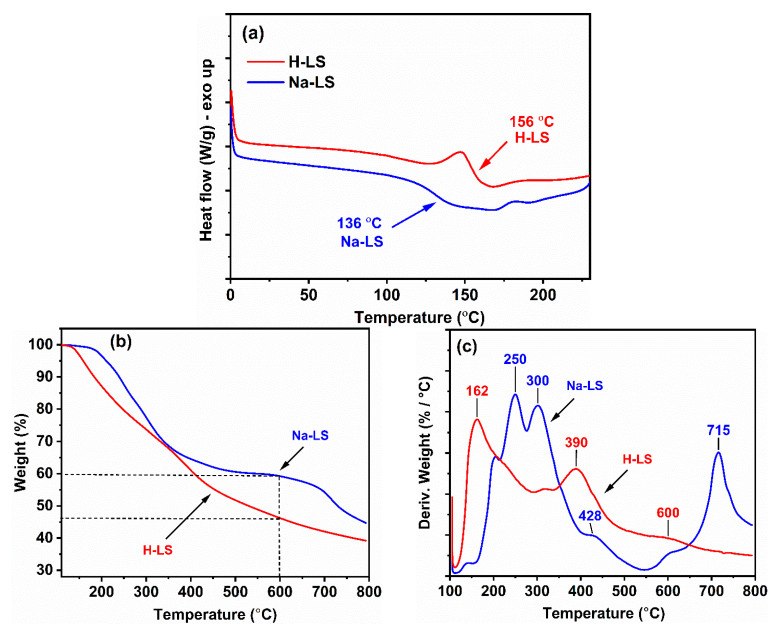
Thermal behavior of lignosulfonate samples (Na-LS and H-LS). (**a**) DSC thermograms, (**b**) thermogravimetric analysis (TGA) curves, and (**c**) the first derivative of the TGA (DTG) curves.

The data on functional groups, apparent Mw, and the chemical structures of lignosulfonate samples discussed above are critical for understanding the thermal behavior of lignin. Figure 6a demonstrates the difference in DSC curves and glass temperature (T_g_) between Na-LS and H-LS samples. The T_g_ value of H-LS was clearly higher than that of Na-LS. This is because, as shown in the FTIR, XPS, Scheme 1, and Figure 3b H-LS contained COOH and SO_3_H groups, which facilitated hydrogen bonding between lignin, constructing aggregation and reducing thermal mobility. In contrast, the presence of the negative charge in Na-LS (COO^–^ and SO_3_^–^) causes less aggregation due to electrostatic repulsion and enhanced thermal mobility. As expected, the T_g_ of H-LS was also greater than that of the kraft lignin samples (150 °C for SKL and 155 °C for HKL [29]). According to GPC and ^31^P NMR, the H-LS had a greater M_w_, more polymeric chains, and more aliphatic OH groups than the SKL and HKL, resulting in less flexibility and more hydrogen bonding between the lignin polymers, which led to reduced thermal mobility. Surprisingly, although having a significantly greater Mw than kraft lignin, the difference in T_g_ between H-LS and kraft lignin is quite insignificant. This is explained by the fact that lignosulfonate has significantly more β-*O*-4′ ether bonds than kraft lignin (Table 3), which enhances the flexibility and mobility of the lignin chain [50] and decreases the T_g_.

The thermal degradation behavior of lignosulfonate samples was also investigated using thermogravimetric analysis (TGA) and its first derivative (DTG). Figure 6a shows the weight loss of lignosulfonate as the temperature increases from around 100 °C to 800 °C, whereas Figure 6b depicts the rate of change in weight with the corresponding temperature of degradation. Interestingly, the pattern of the TGA and DTG graphs of Na-LS were identical to those of Na-LS samples reported in the literature [21,31], whereas the pattern of H-LS graphs was similar to that of ammonium-LS [21]. The TGA curves (Figure 6a) clearly reveal that at the same temperature, H-LS degraded faster or easier than Na-LS, which is most likely owing to the acid groups in H-LS, such as S_3_OH and COOH, which can catalyze lignosulfonate sample decomposition. Likewise, DTG curves (Figure 6b) reveal that H-LS exhibited lower peak temperatures compared to Na-LS. The greatest weight loss for Na-LS occurs at roughly 250–300 °C, 428 °C, and 715 °C, whereas the greatest weight loss for H-LS occurs at around 162 °C, 390 °C, and 600 °C. The initial weight losses at low temperatures, around 100 °C, corresponds to lignin dehydration and the elimination of bound water from the lignin sample [21,31]. In addition, the peak temperatures of H-LS at 162 °C and Na-LS around 250–300 °C were linked to the decomposition of polysaccharides impurities, particularly hemicelluloses. As previously demonstrated by ^31^P and ^1^H–^13^C HSQC NMR results, lignosulfonate typically includes a large amount of carbohydrates impurity bound to the lignin polymer [44,47]. Therefore, the huge weight loss at the beginning is most likely due to their decomposition. Moreover, the TG/DTG results are consistent with the observation that lignin-containing xylose units display a pronounced DTG peak at roughly 200–270 °C [31]. Additionally, it was revealed that the pronounced peak of the pure xylan DTG curve was regarded as its greatest degradation rate, occurring at roughly 280 °C [51]. H-LS appears to have a substantially lower temperature for the maximum decomposition rate of hemicellulose (162 °C) than Na-LS (250–300 °C) because it features acidic functional groups (–SO_3_H, –COOH, –OH). It has been revealed that the presence of acidic moieties, particularly SO_3_H, can accelerate the decomposition of bonds in hemicellulose molecules, resulting in the release of xylose or xylo-oligosaccharides, as well as hydrolysis of the β-1,4-glycosidic bonds in cellulose [52]. Further, it is apparent that H-LS has a lower value than Na-LS for the degradation between 390–715 °C, which is likely owing to the similar self-catalyzing effect of H-LS. The degradation between 350 and 400 °C is related to the dehydrogenation of the lignin side chain, whereas degradation over 400 °C is due to aromatic ring saturation, the fracture of stable C–C bonds, and the liberation of gaseous products, such as H_2_O, CO_2_, and CO [53,54]. Additionally, methane was mostly produced when methoxy groups were cracked between 400 and 550 °C [31,53]. In addition, the breaking of the C–O–C and C=O bonds in lignin caused it to emit CO_2_ at 340 °C and 700 °C and CO at temperatures above 600 °C [31]. Theoretically, it has been observed that the decomposition of formic acid (COO^–^) was more difficult than the decomposition of formic acid (COOH) to produce CO or CO_2_, which was consistent with the DTG results, showing that Na-LS had a higher peak temperature of decomposition of C=O bonds (715 °C) than H-LS (600 °C) because Na-LS had a higher concentration of COO-, while H-LS had a higher concentration of COOH [55].

## 3. Experimental

### 3.1. Materials

Softwood sodium lignosulfonate recovered from sulfite pulping liquor was supplied by Yunjinglinzhi Co., Ltd. (Kunming, China). Amberlite^®^ IR-120 (hydrogen form) ion exchange resin, anhydrous DMF (99.8%), DMF-d7 (99.5 at% deuterium), anhydrous pyridine (99.8%), N-hydroxy-5-norbornene-2,3-dicarboxylic acid imide (NHND), chromium (III) acetylacetonate (99.99%), and 2-chloro-4,4,5,5-tetramethyl-1,2,3-dioxaphospholane (TMDP) were purchased from Sigma Aldrich, USA.

### 3.2. Ion Exchange Procedure

The ion-exchange of Na-LS was carried out by the procedures in previous research [17]. In brief, Na-LS was transformed into H-LS by passing an aqueous solution of Na-LS (25 g/L) through a column packed with 100 g of Amberlite^®^ IR-120 cation exchange resin. The pH of the solution decreased from 7.72 to 1.57, showing that the cation was successfully exchanged. The ion-exchanged solution was freeze-dried (lyophilized) for several days. The sample was ground into a fine powder prior to the subsequent analysis.

### 3.3. Attenuated Total Reflection-FTIR (ATR-FTIR) Spectroscopy

ATR-FTIR measurements were performed using a Bruker Alpha FTIR (Bruker Optics GmbH, Ettlingen, Germany) to compare the functional groups of Na-LS and H-LS samples. The measurements of the powder samples were repeated with 32 scans with a resolution of 4 cm^−1^ and a range of 400–4000 cm^−1^.

### 3.4. Elemental Analysis and Ash Content

The elemental compositions of lignosulfonate samples (Na-LS and H-LS) were determined using a Thermo Scientific™ Flash 2000 elemental analyzer. The absolute contents of C, H, N, and S were measured, and the oxygen (O) content was calculated as 100% minus the total C, H, and S content. The ash content of the samples was evaluated gravimetrically after combustion at 525 °C [56]. Before measurement, the sample was dried in an oven at 105 °C for 3 h. Following that, approximately 0.5 g of moisture-free sample was weighed and put in a muffle furnace at 525 ± 25 °C for 4 h. Then, samples were cooled to room temperature in a desiccator. After samples were weighed on an analytical scale, the percentage of ash was calculated as follows:

(1) $$Ash\;content\;\left(\%\right)=\frac{A}{B}\times 100$$

where *A* denotes the weight of the ash (g) and *B* denotes the weight of the test specimen (moisture-free) (g).

### 3.5. X-ray Photoelectron Spectroscopy (XPS) Analysis

An XPS system (Nexsa, Thermo Fisher Scientific, Waltham, MA, USA) was used to determine the chemical compositions of Na-LS and H-LS samples at surfaces. XPS survey scans at 80 eV and 1 eV steps were used to investigate the Na_1s_, O_1s_, N_1s_, C_1s_, and S_2p_ regions, whereas high-resolution scans at 50 eV and 0.1 eV steps were utilized to identify specific chemical bonding of the samples using a deconvolution approach with a Gaussian function.

### 3.6. Gel Permeation Chromatography (GPC) Analysis

Apparent molecular weight (M_w_) and polydispersity of the lignosulfonate samples were determined by aqueous GPC analysis [36,57]. The experiments were conducted using a Waters Alliance e2695 GPC system (Milford, MA, USA) outfitted with a refractive index (RI) detector, ultrahydrogel 120, ultrahydrogel 250, and ultrahydrogel 500 columns, and column oven set at 35 °C. Pullulans from 6300 to 334,000 Da and poly(ethylene glycol) (PEG) from 106 to 26,300 Da were employed as calibration standards. Briefly, 3 mg of each sample was dissolved in 1 mL of 0.02 N NaNO_3_ and then filtered through a 0.45 µm PTFE syringe filter. Furthermore, 0.02 N NaNO_3_ aqueous solution was used as the eluent at a flow rate of 0.8 mL/min.

### 3.7. Quantitative ^31^P Nuclear Magnetic Resonance (NMR) Spectroscopy

The hydroxyl groups of lignosulfonate samples were quantitated using ^31^P NMR spectroscopy based on a method described in the literature [17]. After converting the Na-LS sample into its acidic form (H-LS), the dry H-LS sample (25 mg) was dissolved in 338 mL of anhydrous DMF and DMF-d7 (3.5:1) solvent mixture. Subsequently, 75 mL of anhydrous DMF containing NHND (~18 mg/mL) as the internal standard and 75 mL of anhydrous pyridine containing chromium (III) acetylacetonate (~5.7 mg/mL) as the relaxation agent were added. After that, 75 mL of phosphitylation reagent (TMDP) was added to the mixture, stirred, and then transferred into an NMR tube for immediate analysis. For comparison, SKL and HKL samples were prepared for quantitative ^31^P NMR analysis using a standard procedure [14]. All ^31^P NMR experiments were performed using a Bruker Avance Neo 700 MHz NMR spectroscopy. An inverse-gated decoupling pulse with the following parameters were used to acquire quantitative ^31^P NMR spectra for determining the amount of hydroxyl groups in all lignin samples: 512 scans, 10 s relaxation delay, ~1.4 s acquisition time, 15 μs pulse width, and 90° pulse angle. The chemical shift of each phosphitylation product was calibrated by attributing a strong peak at 132.2 ppm to the TMDP + H_2_O product [14].

### 3.8. ^1^H–^13^C Heteronuclear Single Quantum Coherence (HSQC) NMR Spectroscopy

2D HSQC spectroscopy was used to quantify the number of linkages and units in the lignosulfonate sample, represented as a number per 100 aromatic units. Two solvents were used for HSQC NMR analysis, DMSO-d_6_ and D_2_O. H-LS sample (40 mg) was dissolved in 750 μL of DMSO-d_6_ [43] and 500 μL of D_2_O [48]. NMR spectra were acquired using Bruker’s “hsqcetgpsisp2.2” pulse program with the following parameters: 32 scans, 1.5 s relaxation delay, ~0.1 s acquisition time, 16.5 s pulse width, and 90° pulse angle. HSQC cross-signals were assigned based on the literature [43].

### 3.9. Differential Scanning Calorimetry (DSC) Analysis

A DSC (Discovery 25, TA instruments, New Castle, DE, USA) was used to measure the glass transition temperature (T_g_) of lignosulfonate samples (Na-LS and H-LS). A dried lignin sample (5 mg) was enclosed in an aluminum pan and covered with a lid. The sample was annealed by heating to 105 °C at a rate of 10 °C/min, then isothermally conditioning for 30 min before cooling to 0 °C. DSC thermograms were then recorded by continuously raising the temperature to 250 °C at a rate of 10 °C/min [29].

### 3.10. Thermogravimetric Analysis

Thermogravimetric analysis (TGA) was performed using a thermal analysis system (Discovery SDT 650, TA instruments). A total of 10 mg of lignosulfonate samples (Na-LS and H-LS) were looked at to evaluate their thermal degradation characteristics. To remove residual moisture, each sample was first heated to 105 °C and kept for 10 min. The sample was then heated from 105 °C to 800 °C at a rate of 10 °C/min under a nitrogen flow of 25 mL/min [29,58].

## 4. Conclusions

In conclusion, Na-LS was successfully converted to H-LS via ion exchange chromatography, as evidenced by changes in color and pH value, drastically reduced ash content, and FTIR and XPS analysis. H-LS exhibited a greater apparent M_w_ than Na-LS because of differences in their sulfonic and carboxylic groups, which impact GPC measurement. In addition, H-LS had a greater M_w_ than the kraft lignin samples (SKL and HKL), indicating that lignosulfonate included more polymeric chains because of the difference in their pulping processes. Accordingly, H-LS showed less aromatic/phenolic OH and much more native side chains, such as β-*O*-4 units, than SKL and HKL, as demonstrated by ^31^P and ^1^H–^13^C HSQC NMR results. In addition, NMR studies showed that the typical carbohydrate impurity remained prominent in the lignosulfonate sample. Thermal investigation revealed that H-LS had a higher T_g_ than Na-LS. This is because H-LS may readily form aggregation owing to hydrogen bonding through its aliphatic OH, as well as its carboxylic and sulfonic acid groups. However, Na-LS may struggle to form aggregation due to electrostatic repulsion. Moreover, H-LS had somewhat higher T_g_ than SKL and HKL due to the H-LS having a much greater M_w,_ but at the same time also having a considerable flexible linkage, such as the β-*O*-4 unit. In addition, H-LS degraded more rapidly than Na-LS because acidic functional groups, notably sulfonic acid, can act as catalysts to facilitate degradation reactions, such as the decomposition of hemicellulose and the breakdown of chemical bonds in lignin polymer.

## Figures and Tables

**Table 1:** Effect of ion exchange treatment on certain parameters, including ash content and elemental composition.

Sample	C (%)	H (%)	N (%)	S (%)	O (%)	O/C Ratio	Ash Content (%)	SO_3_Na/SO_3_H Density * (mmol/g)	Empirical Formula
Na-LS	42.26	4.55	1.04	6.85	26.83	0.63	18.46 ± 2.1	2.14	C_3.52_H_4.55_N_0.07_S_0.21_O_1.68_
H-LS	48.98	5.46	1.07	7.59	35.46	0.72	1.44 ± 0.1	2.37	C_4.08_H_5.46_N_0.08_S_0.24_O_2.22_

* Calculated from the S content.

**Table 2:** Hydroxyl group content of lignin samples measured by ^31^P NMR spectroscopy.

OH Content	H-LS (mmol/g)	SKL (mmol/g) *	HKL (mmol/g) *
Aliphatic OH	2.44	2.27	1.14
Aromatic/Phenolic OH	1.03	3.52	3.75
Syringyl OH	0.00	0.17	1.52
Condensed OH/ other C5-substituted	0.39	1.26	1.00
Guaiacyl OH	0.56	1.86	0.98
*p*-Hydroxyphenyl OH	0.08	0.23	0.25
Carboxylic OH	0.20	0.32	0.48

* Data on OH content were acquired from previous research [29].

**Table 3:** Relative quantification of side chain units identified in lignin samples.

Lignin Units	Abundance (per 100 Aromatic Units)			
	H-LS (DMSO-d_6_)	H-LS (D_2_O)	SKL *	HKL *
(A) Sulfonated β-*O*-4′	41.4	38.9	-	-
(*A*) β-aryl ether (β-*O*-4′)	1.2	-	7.2	3.4
(B) Phenyl coumaran (β-5′)	4.3	3.2	2.3	0
(C) Pinoresinols (β–β′)	7.4	6.6	3.7	4.5
S/G	0	0	0	1.8

* Data on the abundance of lignin units were obtained from previous research [29].

## Data Availability

Data is available on request.

## Sample Availability

Samples of all compounds are available from the authors.
